# Assessment of the susceptibility status of *Aedes aegypti* (Diptera: Culicidae) populations to pyriproxyfen and malathion in a nation-wide monitoring of insecticide resistance performed in Brazil from 2017 to 2018

**DOI:** 10.1186/s13071-020-04406-6

**Published:** 2020-10-27

**Authors:** Kauara Brito Campos, Ademir Jesus Martins, Cynara de Melo Rodovalho, Diogo Fernandes Bellinato, Luciana dos Santos Dias, Maria de Lourdes da Graça Macoris, Maria Teresa Macoris Andrighetti, José Bento Pereira Lima, Marcos Takashi Obara

**Affiliations:** 1 Coordenação Geral de Vigilância de Aboviroses, Secretaria de Vigilância em Saúde, Ministério da Saúde, Edifício PO 700, SRTV 702, Via W 5 Norte, Brasília/Distrito Federal, CEP 70723-040 Brazil; 2 Laboratório de Parasitologia Médica e Biologia de Vetores, Faculdade de Medicina, Universidade de Brasília, Campus Universitário Darcy Ribeiro, Asa Norte, Brasília/Distrito Federal, CEP 70910-900 Brazil; 3 Laboratório de Fisiologia e Controle de Artrópodes Vetores, Instituto Oswaldo Cruz, Rua Francisco Manuel no 102, Bairro Benfica, Rio de Janeiro/Rio de Janeiro State, CEP 20911-270 Brasil; 4 Laboratório de Entomologia Aplicada, Superintendência de Controle de Endemias, Secretaria de Estado da Saúde de São Paulo, Avenida Santo Antônio no 1627, Bairro Somenzari, Marília/São Paulo, CEP 17506-970 Brasil

**Keywords:** Arboviruses, *Aedes aegypti*, Insecticide resistance, Juvenile hormones, Organophosphate insecticides

## Abstract

**Background:**

Chemical mosquito control using malathion has been applied in Brazil since 1985. To obtain chemical control effectiveness, vector susceptibility insecticide monitoring is required. This study aimed to describe bioassay standardizations and determine the susceptibility profile of *Ae. aegypti* populations to malathion and pyriproxyfen, used on a national scale in Brazil between 2017 and 2018, and discuss the observed impacts in arbovirus control.

**Methods:**

The diagnostic-doses (DD) of pyriproxyfen and malathion were determined as the double of adult emergence inhibition (EI) and lethal doses for 99% of the Rockefeller reference strain, respectively. To monitor natural populations, sampling was performed in 132 Brazilian cities, using egg traps. Colonies were raised in the laboratory for one or two generations (F1 or F2) and submitted to susceptibility tests, where larvae were exposed to the pyriproxyfen DD (0.03 µg/l) and adults, to the malathion DD determined in the present study (20 µg), in addition to the one established by the World Health Organization (WHO) DD (50 µg) in a bottle assay. Dose-response (DR) bioassays with pyriproxyfen were performed on populations that did not achieve 98% EI in the DD assays.

**Results:**

Susceptibility alterations to pyriproxyfen were recorded in six (4.5%) *Ae. aegypti* populations from the states of Bahia and Ceará, with Resistance Ratios (RR_95_) ranging from 1.51 to 3.58. Concerning malathion, 73 (55.3%) populations distributed throughout the country were resistant when exposed to the local DD 20 µg/bottle. On the other hand, no population was resistant, and only 10 (7.6%) populations in eight states were considered as exhibiting decreased susceptibility (mortality ratios between 90 and 98%) when exposed to the WHO DD (50 µg/bottle).

**Conclusions:**

The feasibility of conducting an insecticide resistance monitoring action on a nation-wide scale was confirmed herein, employing standardized and strongly coordinated sampling methods and laboratory bioassays. Brazilian *Ae. aegypti* populations exhibiting decreased susceptibility to pyriproxyfen were identified. The local DD for malathion was more sensitive than the WHO DD for early decreased susceptibility detection. 
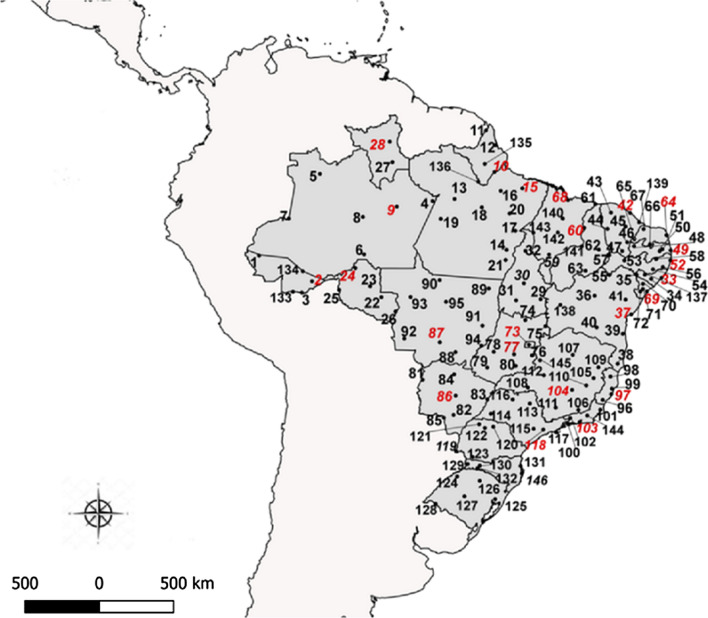

## Background

In recent decades, the incidence of *Aedes*-borne diseases, such as dengue, Zika, chikungunya and yellow fever, has increased significantly worldwide [1]. Actions against the *Aedes* (*Stegomyia*) *aegypti* (Linnaeus, 1762) are mainly based on chemical and mechanical controls aiming to reduce infestation, while social mobilization, environmental management and legislation protections seeking to maintain environments free of larval breeding sites are also applied. Controlling the insect in its immature phases (egg, larva and pupa) is more feasible, since development occurs in specific and restricted locations, unlike the adult phase, which may be dispersed throughout various environments. The most effective form of vector control is environmental management involving mechanical reservoir removal, although arbovirus transmission blocking usually comprises chemical insecticide applications, aiming at rapidly reducing mosquito populations [2].

The Brazilian Ministry of Health (MoH) provides insecticides pre-qualified by the World Health Organization (WHO) to all Brazilian states for the chemical control of *Ae. aegypti*. This process ensures that the entire country employs trusted products concerning environmental safety, toxicity and effectiveness [3]. In addition, the Brazilian MoH evaluates all compounds under local conditions prior to purchases. The application of larvicides by public agents is recommended in domestic reservoirs that cannot be covered or eliminated, every two months. In addition, spatial insecticide application cycles are recommended whenever arbovirus transmission occurs in a given area [4]. Thus, public health actions used to control *Ae. aegypti* in Brazil consume an expressive amount of insecticides each year, considering, for example, that about 4136 Brazilian municipalities registered dengue cases from 2014 to 2017 [5].

With the intensive and continuous deployment of the same active ingredients, resistant individuals in a given population are favorably selected, potentially compromising insecticide efficacy. A rational chemical control strategy should be based on detailed knowledge concerning territorial vector distribution, susceptibility to compounds belonging to different classes and the mechanisms involved in resistance selection, in order to reduce vector infestation levels and consequent arbovirus transmission [6]. Most *Ae. aegypti* populations in America tested for DDT exhibited resistance to this compound (86.7 ± 0.1%). High frequencies of resistant populations were also observed for temephos and deltamethrin (75.7 ± 0.1% and 33 ± 0.1%, respectively). These patterns can be explained by the chronic and frequent use of these insecticides in the continent [7].

In Brazil, insecticide resistance in *Ae. aegypti* was first recorded for the organophosphate (OP) larvicide temephos in populations from the states of Goiás and São Paulo, in 1995 [8]. A few years later, a reduction in temephos resistance was detected in field studies, as well as decreased susceptibility to the adulticide OP fenitrothion and malathion in several *Ae. aegypti* populations throughout the country [9]. In 2001, resistance to the adulticide pyrethroid (PY) cypermethrin was detected in populations from the state of Rio de Janeiro [10]. Within this scenario, the National Dengue Control Programme (PNCD, Portuguese acronym) implemented the National Network for Monitoring the Resistance of *Ae. aegypti* to Insecticides (MoReNAa, Portuguese acronym) in 1999, with the purpose of providing technical support to decisions regarding the chemical control management of *Ae. aegypti*. The MoReNAa Network carried out a systematic insecticide resistance monitoring (IRM) of natural *Ae. aegypti* populations in Brazil to insecticides used in governmental campaigns, in areas considered as either priority or strategic for vector control interventions [11, 12].

Mosquito populations from about 80 cities, including those presenting the highest incidence of dengue cases, most populated, presenting high mosquito infestation indices and all state capitals, were evaluated every two years. Quantitative and qualitative bioassays for larvae and adult resistance detection were performed according to WHO and Centers for Disease Control and Prevention (CDC) methodologies. Biochemical assays for the quantification of enzymatic activity alterations and *kdr* mutation genotyping were employed to investigate the molecular basis of insecticide resistance selection and identify resistance mechanisms. The Network aided in supporting the technical decision concerning insecticide replacement until 2012, when the last monitoring round was carried out [11, 12]. Based on the increasing detection of *Ae. aegypti* populations resistant to temephos, this compound was gradually replaced by insect growth regulators (IGR) since 2009 throughout the entire country, adopting the chitin synthesis inhibitor diflubenzuron, followed by novaluron [9].

The adoption of the IGR pyriproxyfen began in 2014, based on the intention of rotating insecticides presenting distinct modes of action. As a juvenile hormone analogue, this product prolongs the immature stage of the mosquito for up to 20 days, inhibiting the development of imaginal characteristics. A complete metamorphosis is, therefore, compromised, with mortality occurring especially at the pupal stage or leading to the emergence of malformed adults [2]. Some reports indicating resistance to IGR are available, likely because of their recent employment for public health purposes. Some alterations in susceptibility to pyriproxyfen were observed in *Ae. aegypti* populations from Martinique (RR_50_ of 2.2, RR_95_ of 1.9), in 2007 [13] and *Ae. albopictus* from the USA (RR_50_ of 1.8–2.4) [14]. Higher resistance, however, was observed in *Ae. aegypti* from Malaysia (RR_50_ of 6.1) [15] and from the USA (RR_50_ of 38.7, RR_90_ of 81.5), in 2015 [16].

The OP malathion began being employed against adult mosquitoes through ultra-low-volume (ULV) and residual spraying applications in Brazil in 1985. In 1989, it was replaced by fenitrothion for residual spraying, which continued to be used in ULV treatment during the following ten years, when OPs were replaced by PYs for adult control. After years without being used to control *Ae. aegypti* adults, malathion was again adopted alongside the introduction of IGRs for larval control throughout the country since 2009 [9]. OPs are derived from phosphoric acid and its homologs, and their mechanism action acts on the inhibition of the cholinesterase enzyme [2]. Alterations in the susceptibility of *Ae. aegypti* to malathion have already been reported in countries in America, including Brazil [17, 18].

This study was developed with the aim of describing assay standardizations and resistance monitoring of *Ae. aegypti* populations to insecticides used in public health on a national scale in Brazil between 2017 and 2018, discussing the obtained findings. This monitoring was promoted by the Brazilian MoH and was the broadest evaluation ever carried out in a country of continental dimensions, resulting in the evaluation of mosquito populations from 132 cities during 17 months, in which over 137,000 larvae and 131,000 adults were tested. To the best of our knowledge, this is also the largest surveillance round concerning insecticide *Ae. aegypti* resistance monitoring on a global scale.

## Methods

### Study populations

**Fig. 1:**
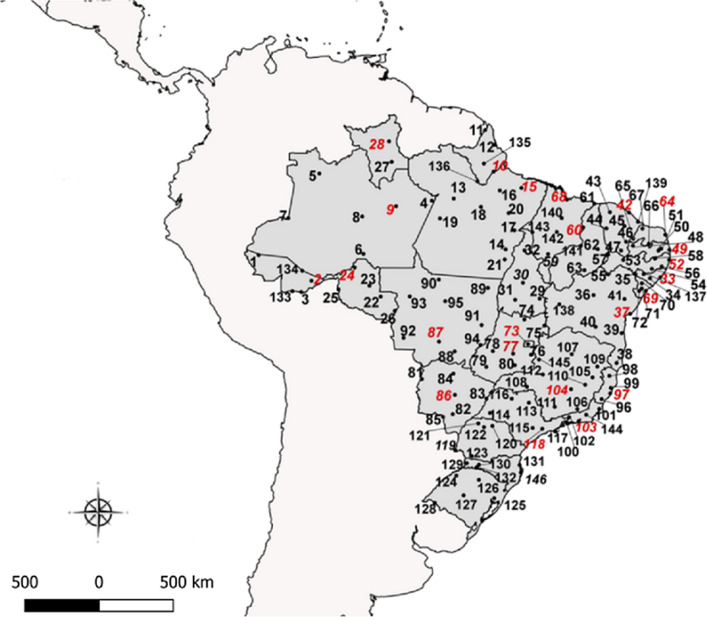
Map of Brazil showing the municipalities participating in the 2017–2018 *Aedes aegypti* pyriproxyfen and malathion susceptibility monitoring round. The numbers in red represent state capitals. The continuous lines in Brazilian territory indicate different states

The sampling points applied herein considered several areas throughout the Brazilian territory, covering a large number of close towns, in urban conglomerates with high population density, as suggested by Chediak et al. [19], preferentially in sites previously evaluated during the 12-year period MoReNAa Network effort, as described by Valle et al. [9]. This proposal was also adjusted considering the operational capacity of the municipal sampling teams, resulting in the selection of 146 cities for *Ae. aegypti* samplings over the course of 17 months (Table 1, Fig. 1). Field *Ae. aegypti* populations were collected by the Endemic Control Agents of each city, using between 100 oviposition traps (ovitraps) in cities with up to 50,000 houses and 300 ovitraps in cities with over 500,000 houses, following the MoReNAa Network methodology [12].

**Table 1:** Brazilian towns participating in the 2017–2018 *Aedes aegypti* pyriproxyfen and malathion monitoring susceptibility round

No.	Lat^a^	Long^b^	State	Town	Nº	Lat^a^	Long^b^	State	Town
1	− 7.36	− 72.67	AC	Cruzeiro do Sul	74	− 13.54	− 48.22	GO	Minaçu
2	− 9.98	− 67.81	AC	Rio Branco	75	− 14.09	− 46.36	GO	Posse
3	− 11.02	− 68.75	AC	Brasiléia	76	− 16.77	− 47.61	GO	Cristalina
4	− 2.63	− 56.74	AM	Parintins	77	− 16.67	− 49.26	GO	Goiânia
5	− 0.14	− 67.08	AM	São Gabriel da Cachoeira	78	− 16.44	− 51.12	GO	Iporá
6	− 7.51	− 63.03	AM	Humaitá	79	− 17.89	− 51.72	GO	Jataí
7	− 4.23	− 69.95	AM	Tabatinga	80	− 17.74	− 49.11	GO	Morrinhos
8	− 4.08	− 63.14	AM	Coari	81	− 19.01	− 57.65	MS	Corumbá
9	− 3.13	− 60.02	AM	Manaus	82	− 22.23	− 54.81	MS	Dourados
10	0.04	− 51.06	AP	Macapá	83	− 20.79	− 51.71	MS	Três Lagoas
11	3.85	− 51.83	AP	Oiapoque	84	− 18.51	− 54.76	MS	Coxim
12	2.50	− 50.94	AP	Calçoene	85	− 22.49	− 55.71	MS	Ponta Porã
13	− 2.44	− 54.72	PA	Santarém	86	− 20.46	− 54.62	MS	Campo Grande
14	− 7.10	− 49.94	PA	Xinguara	87	− 15.57	− 56.07	MT	Cuiabá
15	− 1.46	− 48.49	PA	Belém	88	− 16.47	− 54.63	MT	Rondonópolis
16	− 1.69	− 50.48	PA	Breves	89	− 10.64	− 51.57	MT	Confresa
17	− 5.35	− 49.14	PA	Marabá	90	− 9.87	− 56.09	MT	Alta Floresta
18	− 3.21	− 52.21	PA	Altamira	91	− 14.05	− 52.16	MT	Água Boa
19	− 4.26	− 55.99	PA	Itaituba	92	− 15.23	− 59.34	MT	Pontes e Lacerda
20	− 3.77	− 49.67	PA	Tucuruí	93	− 11.42	− 58.76	MT	Juína
21	− 8.03	− 50.03	PA	Redenção	94	− 15.89	− 52.26	MT	Barra do Garças
22	− 11.43	− 61.44	RO	Cacoal	95	− 11.86	− 55.50	MT	Sinop
23	− 10.44	− 62.48	RO	Jaru	96	− 20.85	− 41.11	ES	Cachoeiro do Itapemirim
24	− 8.77	− 63.83	RO	Porto Velho	97	− 20.32	− 40.32	ES	Vitória
25	− 10.77	− 65.32	RO	Guajará-Mirim	98	− 18.71	− 40.40	ES	Nova Venécia
26	− 12.74	− 60.14	RO	Vilhena	99	− 19.82	− 40.28	ES	Aracruz
27	0.94	− 60.43	RR	Rorainópolis	100	− 23.01	− 44.32	RJ	Angra dos Reis
28	2.82	− 60.67	RR	Boa Vista	101	− 21.75	− 41.33	RJ	Campos dos Goytacazes
29	− 11.63	− 46.82	TO	Dianópolis	102	− 22.51	− 44.09	RJ	Volta Redonda
30	− 10.16	− 48.35	TO	Palmas	103	− 22.88	− 43.23	RJ	Rio de Janeiro
31	-11.73	− 49.07	TO	Gurupi	104	− 19.94	− 43.93	MG	Belo Horizonte
32	− 7.19	− 48.21	TO	Araguaína	105	− 18.85	− 41.95	MG	Governador Valadares
33	− 9.66	− 35.70	AL	Maceió	106	− 21.76	− 43.35	MG	Juiz de Fora
34	− 9.76	− 36.66	AL	Arapiraca	107	− 16.72	− 43.87	MG	Montes Claros
35	− 9.38	− 38.00	AL	Delmiro Gouveia	108	− 19.71	− 47.98	MG	Uberaba
36	− 11.30	− 41.86	BA	Irecê	109	− 17.86	− 41.51	MG	Teófilo Otoni
37	− 13.01	− 38.49	BA	Salvador	110	− 19.53	− 42.62	MG	Coronel Fabriciano
38	− 17.54	− 39.74	BA	Teixeira de Freitas	111	− 21.56	− 45.43	MG	Varginha
39	− 14.79	− 39.27	BA	Itabuna	112	− 18.59	− 46.52	MG	Patos de Minas
40	− 14.21	− 41.67	BA	Brumado	113	− 21.18	− 47.81	SP	Ribeirão Preto
41	− 11.66	− 39.01	BA	Serrinha	114	− 22.12	− 51.39	SP	Presidente Prudente
42	− 3.72	− 38.59	CE	Fortaleza	115	− 23.50	− 47.46	SP	Sorocaba
43	− 3.69	− 40.35	CE	Sobral	116	− 20.81	− 49.38	SP	São José do Rio Preto
44	− 5.18	− 40.67	CE	Crateús	117	− 23.81	− 45.40	SP	São Sebastião
45	− 4.96	− 39.01	CE	Quixadá	118	− 23.57	− 46.57	SP	São Paulo
46	− 6.40	− 38.86	CE	Icó	119	− 25.54	− 54.59	PR	Foz do Iguaçu
47	− 7.21	− 39.32	CE	Juazeiro do Norte	120	− 23.31	− 51.16	PR	Londrina
48	− 6.76	− 38.23	PB	Sousa	121	− 23.08	− 52.46	PR	Paranavaí
49	− 7.15	− 34.87	PB	João Pessoa	122	− 23.42	− 51.94	PR	Maringá
50	− 7.22	− 35.88	PB	Campina Grande	123	− 26.08	− 53.06	PR	Francisco Beltrão
51	− 7.04	− 35.63	PB	Alagoa Grande	124	− 27.87	− 54.48	RS	Santa Rosa
*52*	− 8.06	− 34.89	PE	Recife	125	− 29.95	− 50.99	RS	Gravataí
53	− 8.07	− 39.12	PE	Salgueiro	126	− 28.26	− 52.41	RS	Passo Fundo
54	− 8.89	− 36.49	PE	Garanhuns	127	− 29.69	− 53.81	RS	Santa Maria
55	− 9.40	− 40.50	PE	Petrolina	128	− 30.38	− 56.45	RS	Quaraí
56	− 8.68	− 35.59	PE	Palmares	129	− 26.73	− 53.52	SC	São Miguel do Oeste
57	− 7.58	− 40.50	PE	Araripina	130	− 26.87	− 52.40	SC	Xanxerê
58	− 7.96	− 36.20	PE	Santa Cruz do Capibaribe	131	− 26.91	− 48.66	SC	Itajaí
59	− 6.77	− 43.02	PI	Floriano	132	− 27.11	− 52.62	SC	Chapecó
60	− 5.09	− 42.81	PI	Teresina	133	− 10.94	− 69.56	AC	Assis Brasil
61	− 2.90	− 41.78	PI	Parnaíba	134	− 9.07	− 68.66	AC	Sena Madureira
62	− 7.08	− 41.47	PI	Picos	135	0.78	− 51.95	AP	Pedra Branca do Amapari
63	− 9.02	− 42.69	PI	São Raimundo Nonato	136	− 0.86	− 52.54	AP	Laranjal do Jari
64	− 5.75	− 35.25	RN	Natal	137	− 9.37	− 37.25	AL	Santana do Ipanema
65	− 6.11	− 38.20	RN	Pau dos Ferros	138	− 12.14	− 45.00	BA	Barreiras
66	− 6.59	− 36.77	RN	Jardim do Seridó	139	− 4.57	− 37.77	CE	Aracati
67	− 5.19	− 37.36	RN	Mossoró	140	− 4.23	− 44.78	MA	Bacabal
68	− 2.53	− 44.30	MA	São Luís	141	− 7.53	− 46.04	MA	Balsas
69	− 10.91	− 37.05	SE	Aracaju	142	− 5.51	− 45.24	MA	Barra do Corda
70	− 10.22	− 37.42	SE	Nossa Senhora da Glória	143	− 5.53	− 47.48	MA	Imperatriz
71	− 10.69	− 37.43	SE	Itabaiana	144	− 22.29	− 42.53	RJ	Nova Friburgo
72	− 10.92	− 37.67	SE	Lagarto	145	− 17.22	− 46.88	MG	Paracatu
73	− 15.79	− 47.89	DF	Brasília	146	− 27.59	− 48.55	SC	Florianópolis

^a^Latitude

^b^Longitude

*Note*: State capitals underlined. State acronyms: AC, Acre; AM, Amazonas; AP, Amapá; PA, Pará; RO, Rondônia; RR, Roraima; TO, Tocantins; AL, Alagoas; BA, Bahia; CE, Ceará; PB, Paraíba; PE, Pernambuco; PI, Piauí; RN, Rio Grande do Norte; MA, Maranhão; SE, Sergipe; DF, Distrito Federal; GO, Goiás; MS, Mato Grosso do Sul; ES, Espírito Santo; RJ, Rio de Janeiro; MG, Minas Gerais; SP, São Paulo; PR, Paraná; RS, Rio Grande do Sul; SC, Santa Catarina

To install the traps, houses evenly distributed in a grid pattern with full coverage of the urban territory were selected, in order to include regions presenting different infestation levels, and one trap was installed in a shaded area on the grounds of each selected house. A 0.04% yeast extract solution was used as an attractant for gravid females. In order to facilitate the preparation of this solution in the field, the agents were provided with a 50 ml conical tube containing 6 g of a commercial yeast extract (Arma Zen®; Tetra Gmbh, Melle, Germany). During the trap installation, the tubes were filled with tap water to the 50 ml mark and homogenized. With the aid of plastic Pasteur pipettes, 1 ml of this solution was added to the trap, which was then then filled with tap water to the 300 ml mark. The traps were maintained in the households for 15 days, with one paddle and an attractive solution change at the end of the first week. The paddles containing the eggs were air-dried for 2–3 days prior to being sent to the laboratories.

The samplings were carried out between August 2017 and December 2018, following a staggered schedule so as not to overload the laboratories. Three preferred months were chosen for the samplings in each region of the country, observing the most adequate climatic conditions in order to obtain higher egg densities. The field-collected samples were initially sent to a central entomology laboratory in each respective state, which then confirmed the correct sampling registration at the origin sites and adequate paddle storage. The paddles were then shipped to the Physiology and Arthropod Vector Control Laboratory (Laboratório de Fisiologia e Controle de Artrópodes Vetores, LAFICAVE), at the Oswaldo Cruz Institute (IOC/Fiocruz), Rio de Janeiro/RJ, where the arrivals were recorded, forms were stored and populations labeled with a code known only by the study director, in order to maintain origin confidentiality. Half of the populations remained at the LAFICAVE, while the other half was sent to the Applied Entomology Laboratory (Laboratório de Entomologia Aplicada, LEnA), at the Endemic Control Superintendence (Superintendência de Controle de Endemias, SUCEN), Marília, SP. *Aedes aegypti* specimen sorting, colony maintenance and bioassays were performed by the LAFICAVE and LEnA laboratories.

### Mosquito rearing

Paddles containing eggs were submerged in dechlorinated water and hatched larvae were transferred to basins (33 × 24 × 8 cm) containing 1 l of dechlorinated water and 100 mg of fish food (TetraMin®, Tetra Marine Granules; Tetra Gmbh, Melle, Germany) added every 3 days. The resulting adult *Ae. aegypti* mosquitoes were identified to the species level and sorted sex, with 500 females and 500 males maintained in cylindrical carton cages (16 cm in diameter × 18 cm high), where a 10% sucrose solution was offered *ad libtum.* When the number of females were insufficient for producing an F1 generation (less than 100 females), new field collections were requested.

In order to produce eggs for the next generation, females were additionally fed blood from guinea pigs (*Cavia Porcellus* - Linnaeus, 1758) 3 days post-emergence. Alternatively, females were offered to feed on citrated rabbit blood through a Hemotek reservoir membrane feeder (Discovery Workshops, Accrington, UK), containing 6 ml of blood covered with a parafilm membrane, sealed with a rubber ring, at 37 °C for 1 h. F1 generation mosquitoes were employed in the bioassays, although an F2 generation was required whenever the number of F1 generation individuals to perform all larvae and adult assays was insufficient.

Insectaries were maintained under controlled temperature (26 ± 2 °C) and humidity (70 ± 10%) following the Fiocruz biosafety manual for vector insectaries and infectories [20]. About 50 specimens of the parental generation were cryopreserved for the creation of a DNA bank for future genetic analyses. Only male mosquitoes were cryopreserved, eliminating the need to extract the female’s abdomen to prevent possible DNA amplification from spermatozoa present in their spermateca. The Rockefeller [21] reference strain concerning insecticide susceptibility and vigor under laboratory conditions was employed for the determination of diagnostic-doses (DD), and was exposed in parallel in each assay, as an assay quality control. Standardizations of the biological tests performed on both adults and larvae were carried out using this susceptible strain.

### DD estimations

Before the susceptibility evaluations of field *Ae. aegypti* populations, the DD for pyriproxyfen and malathion were estimated, respectively, in larvae and adults, under our local conditions. It is important to note that a WHO reference for a pyriproxyfen DD is still not available so far. The locally established DDs were obtained by dose-response (DR) assays using the Rockefeller strain. The Rockefeller colony maintained at the LEnA was used for the tests in both laboratories.

#### DD estimation for pyriproxyfen

Larval bioassays were conducted with an IGR pyriproxyfen analytical standard (Sigma-Aldrich, Co., St Louis, USA), pre-dissolved in acetone (Sigma-Aldrich) and further diluted in ethanol (Merck, CGaA, Darmstadt, Germany). Following procedures described in the WHO guidelines for larvicide bioassays, with some modifications [22], third-stage larvae (L3 stage) were submitted to a gradient of 13 product concentrations (0.0667 to 0.2337µg/l), where adult emergence inhibition (EI) percentages were evaluated at the end of 7 to 10 days, when all control larvae had emerged into adults. Four replicates comprising 10 L3 larvae each were prepared for each concentration, and an equal number of controls were prepared using only ethanol. The larvae were fed 10 mg of fish food (TetraMin®, Tetra Marine Granules) on the first day and 5 mg on the third day after initial exposure. The assays were followed daily until complete adult emergence in the control group.

Assays were discarded if the EI of the control group was > 10%. If not, they were corrected using the Abbott’s formula when EI ranged between 5% and 10% [22]. Four tests were performed at different times. When pupae began to develop, cups were covered with a mesh to avoid eventual adult escapes. Mortality and adult emergence were recorded when all the specimens under the control condition had emerged. Live adults were considered as those totally free of their exuviae and able to fly when gently touched, and the other individuals were considered dead. The EI were calculated using Probit (Polo-PC, LeOra Software, Berkeley, CA, USA) and logistic regression analyses [23]. Finally, the pyriproxyfen DD was determined as twice the dose that inhibited the emergence of adults in 99% (EI_99_) of Rockefeller larvae exposed to the compound.

#### DD estimation for malathion

To perform the bioassays, aliquots of OP stock solutions at a concentration of 3000 mg/l were prepared from a malathion analytical standard (Sigma-Aldrich) dissolved in acetone (Sigma-Aldrich) and stored at -80 °C. Glass bottles (250 ml) (Wheaton) were coated on the inside with 1 ml of malathion dissolved in acetone at four concentrations (12, 15, 18 and 20 µg/bottle) prepared from the stock solution 24 h before the test. Two bottles per concentration and one control (coated on the inside with 1 ml of acetone only) were employed for each test, with each bottle containing 25 females aged 3–5 days-old. Six tests with each dose were performed, on distinct days. Mosquitoes were exposed to the insecticide for up to 30 min, and mortality rates were recorded every 10 min. The dose that caused 100% mortality in 30 min was considered as the DD, as recommended by the WHO [22]. The DD tests with field populations consisted of 25 females aged 3 to 5 days old gently blown with a Castor aspirator inside the bottles: 4 bottles coated with the malathion DD and 2 controls coated with acetone only. Addition tests were conducted applying the WHO recommended DD (50 µg/bottle) [24]. Three independent assays were performed for each population and using both laboratory-determined and WHO recommended DDs.

### Evaluation of pyriproxyfen susceptibility in field populations

#### First screening with DD

Once DD of the pyriproxyfen was obtained, larvae from each field population (16 replicates of 10 larvae, totaling 160 larvae) were exposed to the IGR DD, while 80 larvae from the same population (8 replicates of 10 larvae) were used as the negative control (ethanol only). In parallel, 80 Rockefeller larvae (8 replicates of 10 larvae) were also exposed to the DD, as the internal control of assay conditions. Only healthy larvae exhibiting normal movement and from the same breeding site were selected for each test. The IGR solutions were prepared from a pyriproxyfen analytical standard (Sigma-Aldrich) pre-dissolved in acetone (Sigma-Aldrich) and further diluted in ethanol (Merck®). Aliquots containing 15 µl of the IGR at a concentration of 100,000 mg/l were prepared and stored at − 80 °C. These aliquots were then used to prepare 5 ml stock solutions at a concentration of 300 mg/l and were stored in a refrigerator for up to 30 days. A new dilution was prepared on the same day of the tests from these stock solutions, at a final concentration from which 1 ml would result in the desired DD in the 250 ml test cups. Each population was tested four independent times. The EI of each population was established as the means of these four assays. A total of 240 larvae from the evaluated field population (including their replicates) were necessary for each dose-diagnostic test, totaling 960 larvae in the four repetitions performed in different rounds. WHO criteria were applied to classify the populations as susceptible, exhibiting suggested resistance or resistant, when EI were ≥ 98%, between 90 and 97.9% and < 90%, respectively [22].

#### Resistance ratio estimation

Field populations not susceptible to pyriproxyfen (EI < 98%) in DD assays were submitted to a DR assay in order to quantify their resistance levels. Larvae were exposed to a range of 10 concentrations (0.008–0.45 µg/l) in four replicates comprising 10 L3 larvae each and four control replicates using ethanol only. The Rockefeller strain was run in parallel, consisting of four replicates, with larvae exposed to the DD only. Mortality and metamorphosis rates were recorded until the emergence of all adults in the control condition. A total of 440 larvae were evaluated in each DR test, including their replicates, requiring 1760 larvae from each field population to perform the repetitions of the four different rounds.

The inhibition of 50% and 95% adult emergence (EI_50_ and EI_95_) of each population were obtained by a probit analysis [25]. Resistance ratios were obtained by dividing the EI (50 and 95) of each population by the equivalent EI of the Rockefeller reference strain. Populations were classified as suggested by Mazzarri & Georghiou [26] into low, moderate, or high resistance respectively for RR_95_ < 5, between 5.0–10.0, and > 10.0.

### Evaluation of malathion susceptibility in field populations

The *Ae. aegypti* populations were tested using adult females, 3 to 5 days post-emergence and not blood-fed, from the F1 or F2 generations. Each test consisted of the exposure of 20 to 25 females per bottle, with 4 bottles coated on the inside with each DD (the DD evaluated herein and 50 µg/bottle) in addition to 2 bottles coated on the inside with acetone only as the negative control. The reference Rockefeller strain was run in parallel with 2 bottles coated with each DD. Mortality rates were recorded every 15 min, and mosquitoes that could not stand, were considered dead. Mortality rates for the replicates of each DD were calculated at the diagnosis time (30 min) in each assay. A total of 4 bioassays were performed for each population, and the final result considered the mean mortality of these bioassays. A total of 1000 females from each field population were used to carry out four different rounds of these tests, comprising 250 females in each, including replicates.

The DD and DR assays for both the IGR and adulticide compounds were performed under test-insectary conditions, with controlled temperature (26 ± 2 °C) and humidity (70 ± 10%).

### Data analysis

The percentages of adult emergence inhibition, lethal doses (LD), their respective confidence intervals (95% CI) and the population slope were calculated by the Polo-PC software, employing a probit analysis [25]. Resistance ratios (RR) were obtained by the quotient between the LD of a population by the Rockefeller reference strain values. Maps were constructed using the QGIZ version 2.18.6 and GIMP version 2.10.14 software packages [23].

## Results

A total of 146 urban Brazilian cities were selected to evaluate *Ae. aegypti* susceptibility/resistance to insecticides current employed in official national campaigns throughout the country (Table 1, Fig. 1), based on a geographical representation proposal. State capitals, international borders and cities exhibiting previous insecticide resistance data were preferentially selected. Appropriate egg sampling was performed in 140 (95.9%) localities. Eggs from 14 (9.6%), however, did not hatch or the number of resulting larvae were insufficient to produce a F1 generation (less than 100 females). Thus, new samplings were carried out in a further six (4.1%) localities. Female numbers remained low even after a second collection and F1 *Ae. aegypti* colonies were raised with less than 100 F0 females for four localities, namely Parintins (Amazonas), Irecê (Bahia), Quixadá (Ceará) and Salgueiro (Pernambuco). A total of 132 *Ae. aegypti* populations (94.3% of the initially planned point collections) were evaluated. The number of *Ae. aegypti* mosquitoes obtained per population ranged from 48 to 2438 females and from 54 to 2563 males. *Aedes albopictus* was present in 59.8% (78/132) of the populations, at 1–419 females and 1–455 male ratios.

Table 2 presents information regarding the geographical origin, number of total and positive paddles (paddles containing eggs), mean egg numbers in positive paddles, total resulting adults for both *Ae. aegypti* and *Ae. albopictus*, adult emergence inhibition (EI) to the IGR larvicide and mortality after exposure to the adulticide organophosphate.

**Table 2:** Evaluation of resistance to pyriproxyfen and malathion in *Aedes aegypti* from Brazil, 2017–2018

N0	Reg	State	Town	Paddles			Adult mosquitoes^a^				Insecticide					
				total	pos^b^	mean eggs in pp^c^	*Ae. aegypti*		*Ae. albopictus*		Pyriproxifen (*Ae. aegypti* larvae)			Malathion (*Ae. aegypti* adults)		
							fem^d^	male	fem^d^	male	EI% cont^e^	EI% (DD 0.03)^f^	EI% cor^g^	Mort% cont^h^	Mort% (DD 20)^i^	Mort% (DD 50)^j^
1	**N**	**AC**	**Cruzeiro do Sul**	196	72	73.3	601	793	0	0	0.94	100.0	NN	0.00	**58.2**	99.3
2	**N**	**AC**	**Rio Branco**	294	188	83.8	2377	2533	0	0	1.61	100.0	NN	0.00	**75.3**	99.0
3	**N**	**AC**	**Brasiléia**	100	43	67.7	734	814	0	0	0.31	99.5	NN	0.00	**71.4**	99.4
4	**N**	**AM**	**Parintins**	196	39	69.5	90	54	96	91	0.94	100.0	NN	0.00	**75.3**	100.0
5	N	AM	São Gabriel da Cachoeira	200	46	101.7	423	383	0	0	3.25	100.0	NN	0.00	100.0	100.0
6	**N**	**AM**	**Humaitá**	200	67	28.9	696	690	0	0	1.88	100.0	NN	0.00	**57.0**	99.7
7	**N**	**AM**	**Tabatinga**	172	50	64.3	472	504	0	0	0.00	100.0	NN	0.00	**68.7**	98.7
8	**N**	**AM**	**Coari**	196	70	WI	253	216	0	0	0.63	100.0	NN	0.00	**63.3**	98.3
9	**N**	**AM**	**Manaus**	512	207	48.8	1021	1047	187	98	1.50	100.0	NN	0.00	**41.0**	98.0
10	**N**	**AP**	**Macapá**	265	79	32.5	296	209	0	0	0.31	100.0	NN	0.00	**80.6**	100.0
11	**N**	**AP**	**Oiapoque**	200	28	33.1	WI	WI	WI	WI	2.81	100.0	NN	0.00	**93.3**	100.0
12	**N**	**AP**	**Calçoene**	74	14	45.3	207	178	0	0	1.56	100.0	NN	0.00	**76.4**	98.8
13	**N**	**PA**	**Santarém**	302	87	43.7	362	382	102	78	5.00	100.0	NN	0.00	**85.3**	98.8
14	**N**	**PA**	**Xinguara**	202	35	107.0	515	501	0	0	0.94	99.5	NN	0.00	**75.5**	99.1
15	**N**	**PA**	**Belém**	600	361	55.0	1751	1787	419	342	1.33	99.5	NN	0.00	**75.0**	98.6
16	**N**	**PA**	**Breves**	202	26	101.7	516	512	4	7	1.87	99.5	NN	0.00	**83.2**	97.2
17	**N**	**PA**	**Marabá**	300	96	77.0	503	500	0	1	2.75	99.4	NN	0.00	**79.5**	100.0
18	**N**	**PA**	**Altamira**	304	103	66.9	526	503	4	28	3.44	99.1	NN	0.00	**88.1**	99.4
19	**N**	**PA**	**Itaituba**	200	102	96.2	426	392	416	280	2.19	98.9	NN	0.00	**35.5**	99.4
20	**N**	**PA**	**Tucuruí**	198	93	79.4	504	501	219	158	3.43	98.9	NN	0.00	**80.1**	96.6
21	**N**	**PA**	**Redenção**	200	29	88.7	384	321	1	1	0.63	98.3	NN	0.00	**65.8**	98.5
22	N	RO	Cacoal	196	52	29.3	329	414	0	8	0.00	100.0	NN	0.00	100.0	100.0
23	N	RO	Jaru	200	85	91,9	1843	1607	141	72	0.50	100.0	NN	0.00	99.0	100.0
24	N	RO	Porto Velho	300	116	54.0	1222	1042	257	167	0.75	99.9	NN	0.00	100.0	100.0
25	N	RO	Guajará-Mirim	194	58	44.4	1248	1374	0	0	0.31	99.8	NN	0.00	99.3	100.0
26	N	RO	Vilhena	200	79	57.1	1457	1583	0	0	0.00	99.2	NN	0.00	100.0	100.0
27	**N**	**RR**	**Rorainópolis**	WI	39	54.5	352	198	0	0	0.62	100.0	NN	0.00	**87.4**	100.0
28	**N**	**RR**	**Boa Vista**	300	166	78.4	2293	2428	1	6	0.25	98.8	NN	0.00	**83.0**	100.0
29	N	TO	Dianópolis	204	31	29.1	206	249	0	0	0.00	100.0	NN	0.00	99.3	100.0
30	**N**	**TO**	**Palmas**	288	92	77.7	578	262	12	32	2.25	100.0	NN	0.00	**61.7**	99.7
31	N	TO	Gurupi	208	35	30.1	240	251	0	0	0.63	99.9	NN	0.00	99.3	100.0
32	**N**	**TO**	**Araguaína**	344	129	45.7	501	500	1	1	2.49	98.4	NN	0.00	**63.0**	99.1
33	**NE**	**AL**	**Maceió**	386	102	60.9	496	395	41	20	1.56	100.0	NN	0.00	**92.3**	99.7
34	**NE**	**AL**	**Arapiraca**	296	92	80.2	1128	1007	0	0	0.31	99.1	NN	0.00	**94.1**	99.4
35	**NE**	**AL**	**Delmiro Gouveia**	184	87	37.8	523	309	0	0	5.00	98.6	NN	0.00	**56.9**	99.1
36	NE	BA	Irecê	210	23	17.2	48	59	0	0	0.63	100.0	NN	0.00	99.3	100.0
37	NE	BA	Salvador	878	327	84.7	2264	2349	140	173	0.31	100.0	NN	0.00	100.0	100.0
38	**NE**	**BA**	**Teixeira de Freitas**	220	83	51.8	503	502	0	0	3.44	98.8	NN	0.00	**86.0**	99.1
39	**NE**	**BA**	**Itabuna**	349	155	63.4	505	606	0	2	0.94	**96.5**	NN	0.00	**89.1**	98.1
40	**NE**	**BA**	**Brumado**	220	90	43.4	289	322	1	1	1.56	**91.6**	NN	0.00	**86.8**	99.1
41	**NE**	**BA**	**Serrinha**	204	99	47.0	500	500	0	0	0.63	**85.8**	NN	0.00	**83.1**	98.1
42	**NE**	**CE**	**Fortaleza**	696	269	67,1	1491	1829	80	92	1.94	100.0	NN	0.00	**70.6**	98.3
43	**NE**	**CE**	**Sobral**	300	97	70.8	872	927	0	0	1.88	99.8	NN	0.00	**44.2**	98.5
44	**NE**	**CE**	**Crateús**	100	WI	WI	871	1011	0	0	2.25	99.3	NN	0.00	**31.3**	**97.3**
45	**NE**	**CE**	**Quixadá**	192	34	74.3	76	64	0	0	3.75	**97.7**	NN	0.00	**81.0**	100.0
46	**NE**	**CE**	**Icó**	200	131	70.9	1919	1997	27	10	3.43	**96.1**	NN	0.00	**87.3**	100.0
47	**NE**	**CE**	**Juazeiro do Norte**	300	138	178.2	502	500	0	1	1.56	**95.3**	NN	0.00	**58.8**	99.1
**48**	**NE**	**PB**	**Sousa**	200	63	29.9	405	426	0	0	3.44	100.0	NN	0.00	**75.0**	99.3
**49**	**NE**	**PB**	**João Pessoa**	388	239	50.3	1756	1816	34	31	0.63	100.0	NN	0.00	**64.3**	**91.3**
**50**	**NE**	**PB**	**Campina Grande**	300	91	43.4	1007	1013	0	0	1.25	98.6	NN	0.00	**87.2**	99.7
**51**	**NE**	**PB**	**Alagoa Grande**	200	88	31.1	510	508	0	0	0.63	98.1	NN	0.00	**88.9**	99.4
**52**	**NE**	**PE**	**Recife**	891	455	66.1	731	730	87	68	0.00	100.0	NN	0.00	**97.3**	100.0
53	NE	PE	Salgueiro	224	18	22.9	86	127	0	0	0.31	100.0	NN	0.00	100.0	100.0
54	**NE**	**PE**	**Garanhuns**	219	47	22.6	274	297	0	0	0.94	100.0	NN	0.00	**94.5**	99.1
55	NE	PE	Petrolina	300	29	18.8	126	138	0	0	0.62	100.0	IS	IS	IS	IS
56	**NE**	**PE**	**Palmares**	198	90	74.6	962	877	102	71	0,31	99.8	NN	0.00	**96.0**	100.0
57	**NE**	**PE**	**Araripina**	WI	107	48.9	881	834	0	0	1.88	99.8	NN	0.00	**37.6**	98.8
58	**NE**	**PE**	**Santa Cruz do Capibaribe**	303	144	70.1	511	566	0	0	2.19	98.9	NN	0.00	**93.9**	99.1
59	NE	PI	Floriano	190	56	20.9	757	736	54	29	2.75	100.0	NN	0.00	100.0	100.0
60	NE	PI	Teresina	414	125	44.0	915	1034	360	273	2.00	99.8	NN	0.00	99.7	100.0
61	NE	PI	Parnaíba	251	190	78.3	1950	2191	77	63	0.25	99.6	NN	0.00	98.3	100.0
62	**NE**	**PI**	**Picos**	100	29	54.7	307	299	0	0	6.87	98.4	98.3	0.00	**77.0**	**91.2**
63	**NE**	**PI**	**São Raimundo Nonato**	100	23	20.1	165	191	0	0	2.58	98.3	NN	0.00	**81.1**	**92.9**
64	NE	RN	Natal	400	277	66.0	1761	1847	144	188	0.00	100.0	NN	0.00	100.0	100.0
65	NE	RN	Pau dos Ferros	238	45	59.1	806	854	0	0	0.83	100.0	NN	0.00	99.0	100.0
66	**NE**	**RN**	**Jardim do Seridó**	100	62	74.1	507	507	0	3	3.44	100.0	NN	0.00	**87.2**	99.4
67	NE	RN	Mossoró	298	205	78.6	2012	1858	0	0	1.00	99.9	NN	0.00	99.3	100.0
68	NE	MA	São Luís	406	154	58.0	1882	2148	152	102	1.56	100.0	NN	0.00	99.6	100.0
69	NE	SE	Aracaju	416	196	78.6	2438	2563	32	41	0.31	100.0	NN	0.00	99.3	100.0
70	**NE**	**SE**	**Nossa Senhora da Glória**	214	84	94.6	500	502	0	7	3.75	99.7	NN	0.00	**85.5**	98.4
71	**NE**	**SE**	**Itabaiana**	324	139	44.5	504	503	0	2	1.25	98.4	NN	0.00	**95.0**	99.7
72	**NE**	**SE**	**Lagarto**	328	192	78.2	508	500	0	2	4.00	98.2	NN	0.00	**89.0**	98.2
73	**MW**	**DF**	**Brasília**	291	35	50.8	454	526	6	8	1.25	100.0	NN	0.00	**95.6**	100.0
74	**MW**	**GO**	**Minaçu**	100	33	28.9	174	86	215	178	2.19	100.0	NN	0.00	**71.6**	100.0
75	**MW**	**GO**	**Posse**	200	81	45.6	564	535	237	203	1.25	100.0	NN	0.00	**90.1**	98.2
76	**MW**	**GO**	**Cristalina**	WI	98	54.2	1003	930	0	0	0.31	99.8	NN	0.00	**82.7**	**93.4**
77	**MW**	**GO**	**Goiânia**	604	222	58.3	2211	2129	84	60	3.44	99.4	NN	0.00	**69.7**	98.6
78	MW	GO	Iporá	200	133	82.3	508	509	0	8	0.50	99.1	NN	0.00	98.4	100.0
79	**MW**	**GO**	**Jataí**	214	121	43.7	513	502	0	0	0.75	98.3	NN	0.00	**91.3**	100.0
80	**MW**	**GO**	**Morrinhos**	WI	98	88.5	1375	593	1	0	0.94	98.1	NN	0.00	**68.9**	99.4
81	MW	MS	Corumbá	200	70	45.2	802	1099	0	0	0.00	100.0	NN	0.00	98.3	100.0
82	MW	MS	Dourados	300	126	58.8	1921	2104	6	7	0.00	100.0	NN	0.00	99.3	100.0
83	**MW**	**MS**	**Três Lagoas**	274	80	62.0	919	962	12	13	0.63	100.0	NN	0.00	**97.6**	100.0
84	MW	MS	Coxim	188	43	29.2	172	165	15	30	3.13	100.0	NN	0.00	98.5	99.7
85	**MW**	**MS**	**Ponta Porã**	189	46	43.0	455	453	0	0	4.69	100.0	NN	0.00	**90.7**	99.1
86	MW	MS	Campo Grande	408	67	44.6	663	611	0	0	0.31	99.1	NN	0.00	99.0	100.0
87	**MW**	**MT**	**Cuiabá**	394	28	74.1	2399	2369	62	88	0.31	100.0	NN	0.00	**82.0**	100.0
88	**MW**	**MT**	**Rondonópolis**	900	158	52.0	1207	1300	23	13	0.63	100.0	NN	0.00	**82.5**	100.0
89	**MW**	**MT**	**Confresa**	108	69	111.2	1581	1715	103	121	2.19	100.0	NN	0.00	**62.1**	99.7
90	**MW**	**MT**	**Alta Floresta**	118	56	83.4	1394	1411	246	170	2.18	100.0	NN	0.00	**80.1**	91.6
91	**MW**	**MT**	**Água Boa**	202	WI	WI	518	510	3	7	1.25	99.8	NN	0.00	**92.6**	100.0
92	**MW**	**MT**	**Pontes e Lacerda**	208	WI	WI	534	544	0	0	1.88	99.8	NN	0.00	**84.2**	99.1
93	**MW**	**MT**	**Juína**	132	93	72.8	735	1006	0	0	1.25	99.1	NN	0.00	**94.1**	99.4
94	**MW**	**MT**	**Barra do Garças**	200	101	59.3	503	503	7	34	1.88	98.7	NN	0.00	**88.6**	100.0
95	**MW**	**MT**	**Sinop**	150	17	30.8	102	85	2	0	0.94	98.7	NN	0.00	**88.6**	100.0
96	**SE**	**ES**	**Cachoeiro do Itapemirim**	286	163	61.3	1846	1925	248	293	1.88	100.0	NN	0.00	**46.8**	**94.3**
97	**SE**	**ES**	**Vitória**	448	233	86.3	278	291	9	4	3.00	99.5	NN	0.00	**84.8**	99.7
98	**SE**	**ES**	**Nova Venécia**	192	93	73.5	506	503	17	39	3.44	99.4	NN	0.00	**88.2**	99.1
99	**SE**	**ES**	**Aracruz**	202	WI	WI	500	531	2	13	1.24	98.1	NN	0.00	**93.8**	98.5
100	**SE**	**RJ**	**Angra dos Reis**	323	107	32.1	425	391	119	118	1.25	100.0	NN	0.00	**72.0**	100.0
101	SE	RJ	Campos dos Goytacazes	330	119	47.8	1386	1242	14	8	0.00	100.0	NN	0.00	99.3	100.0
102	**SE**	**RJ**	**Volta Redonda**	296	183	88.1	2140	2235	344	455	4.38	100.0	NN	0.00	**76.2**	100.0
103	**SE**	**RJ**	**Rio de Janeiro**	612	306	61.6	2399	2260	90	82	1.75	100.0	NN	0.00	**83.0**	99.0
104	**SE**	**MG**	**Belo Horizonte**	1,77	935	68.3	2360	2175	93	96	1.25	100.0	NN	0.00	**79.3**	100.0
105	**SE**	**MG**	**Governador Valadares**	288	230	60.2	1731	1916	95	114	2.50	100.0	NN	0.00	**93.3**	100.0
106	SE	MG	Juiz de Fora	404	37	27.2	218	244	46	20	0.00	100.0	NN	0.00	99.0	100.0
107	SE	MG	Montes Claros	396	68	20.9	131	136	0	0	0.94	100.0	NN	0.00	100.0	100.0
108	SE	MG	Uberaba	94	53	35.9	273	289	0	0	0.31	100.0	NN	0.00	98.3	100.0
109	**SE**	**MG**	**Teófilo Otoni**	296	110	29.8	502	502	55	45	4.38	100.0	NN	0.00	**82.3**	99.4
110	**SE**	**MG**	**Coronel Fabriciano**	264	WI	WI	107	103	0	0	1.25	99.2	NN	0.00	**63.5**	99.4
111	**SE**	**MG**	**Varginha**	292	39	19.4	210	191	6	3	4.38	98.4	NN	0.00	**94.7**	99.7
112	**SE**	**MG**	**Patos de Minas**	297	WI	WI	510	504	10	2	0.94	98.1	NN	0.00	**90.2**	99.7
113	**SE**	**SP**	**Ribeirão Preto**	WI	WI	WI	118	166	0	0	3.13	100.0	NN	0.00	**97.5**	100.0
114	**SE**	**SP**	**Presidente Prudente**	WI	WI	WI	521	555	0	0	3.13	100.0	NN	0.00	**97.8**	98.7
115	**SE**	**SP**	**Sorocaba**	WI	WI	WI	500	506	8	15	1.88	100.0	NN	0.00	**97.9**	98.5
116	**SE**	**SP**	**São José do Rio Preto**	WI	WI	WI	130	184	0	0	0.63	99.8	NN	0.00	**95.1**	99.1
117	**SE**	**SP**	**São Sebastião**	WI	WI	WI	515	505	32	2	2.81	99.8	NN	0.00	**87.9**	99.1
118	**SE**	**SP**	**São Paulo**	WI	WI	WI	500	529	0	0	0.63	99.5	NN	0.00	**86.1**	99.5
119	**S**	**PR**	**Foz do Iguaçu**	298	72	61.6	947	878	11	7	0.00	100.0	NN	0.00	**86.7**	100.0
120	**S**	**PR**	**Londrina**	400	180	77.4	1537	1955	37	59	1.25	100.0	NN	0.00	**78.0**	100.0
121	**S**	**PR**	**Paranavaí**	200	50	67.5	502	512	0	0	2.25	100.0	NN	0.00	**91.1**	98.8
122	**S**	**PR**	**Maringá**	400	149	60.4	504	500	0	3	2.50	100.0	NN	0.00	**78.4**	**96.5**
123	**S**	**PR**	**Francisco Beltrão**	194	29	31.3	241	241	0	0	0.00	99.1	NN	0.00	**93.7**	99.7
124	**S**	**RS**	**Santa Rosa**	200	116	76.3	164	123	3	0	3.85	100.0	NN	0.00	**90.6**	100.0
125	**S**	**RS**	**Gravataí**	292	175	58.8	584	776	0	42	3.13	99.7	NN	0.00	**94.5**	98.8
126	**S**	**RS**	**Passo Fundo**	300	164	36.6	528	668	0	1	1.25	99.7	NN	0.00	**97.3**	98.5
127	**S**	**RS**	**Santa Maria**	300	180	101.0	524	502	0	2	3.08	98.8	NN	0.00	**57.8**	98.8
128	**S**	**RS**	**Quaraí**	199	20	34.6	219	210	0	0	4.36	98.5	NN	0.00	**94.0**	100.0
129	**S**	**SC**	**São Miguel do Oeste**	200	51	46.1	664	637	18	6	0.31	99.8	NN	0.00	**78.7**	100.0
130	**S**	**SC**	**Xanxerê**	200	89	44.8	1000	1323	0	0	0.00	99.6	NN	0.00	**73.3**	99.1
131	**S**	**SC**	**Itajaí**	300	219	45.4	2074	2247	30	33	1.56	99.5	NN	0.00	**87.7**	100.0
132	S	SC	Chapecó	300	143	99.9	1050	1022	0	3	2.50	98.4	NN	0.00	98.1	100.0

*Notes:* Results are presented in percentage of adult emergence inhibition (EI) or mortality to diagnostic doses of the insecticides. ^a^Adult mosquitoes: total of adult mosquitoes (*Ae. aegypti* and *Ae. albopictus*) from rearing of each field population (F0 generation). ^b^pos: positive paddle. ^c^mean eggs in pp: mean eggs in positive paddle. ^d^fem: female. ^e^EI% cont: Percentage of adult emergence inhibition in control goup. ^f^EI%: Percentage of adult emergence inhibition, 0.03 µg/l Diagnostic Dose (DD). ^g^EI% cor: EI corrected by Abbott's formula if necessary (when was between 5% and 10%). ^h^Mort% cont: Percentage of mortality in control goup. ^i^Mort%: Percentage of mortality, 20 µg/l DD. ^j^Mort%: Percentage of mortality, 50 µg/l DD (WHO, 2016). Underlined: State capitals. Bold: non-susceptible population (EI or mortality below 98%) (WHO, 2016). WI: Without information. IS: Insufficient sample quantity to perform the assay. NN: Correction wasn't necessary. Regions acronyms: N: North, NE: Northeast, CW: Mid-West, SE: South-East, S: South. States acronyms: AC: Acre, AM: Amazonas, AP: Amapá, PA: Pará, RO: Rondônia, RR: Roraima, TO: Tocantins, AL: Alagoas, BA: Bahia; CE: Ceará, PB: Paraíba, PE: Pernambuco, PI: Piauí, RN: Rio Grande do Norte, MA: Maranhão, SE: Sergipe, DF: Distrito Federal, GO: Goiás, MS: Mato Grosso do Sul, ES: Espírito Santo, RJ: Rio de Janeiro, MG: Minas Gerais, SP: São Paulo, PR: Paraná, RS: Rio Grande do Sul, SC: Santa Catarina.

**Fig. 2:**
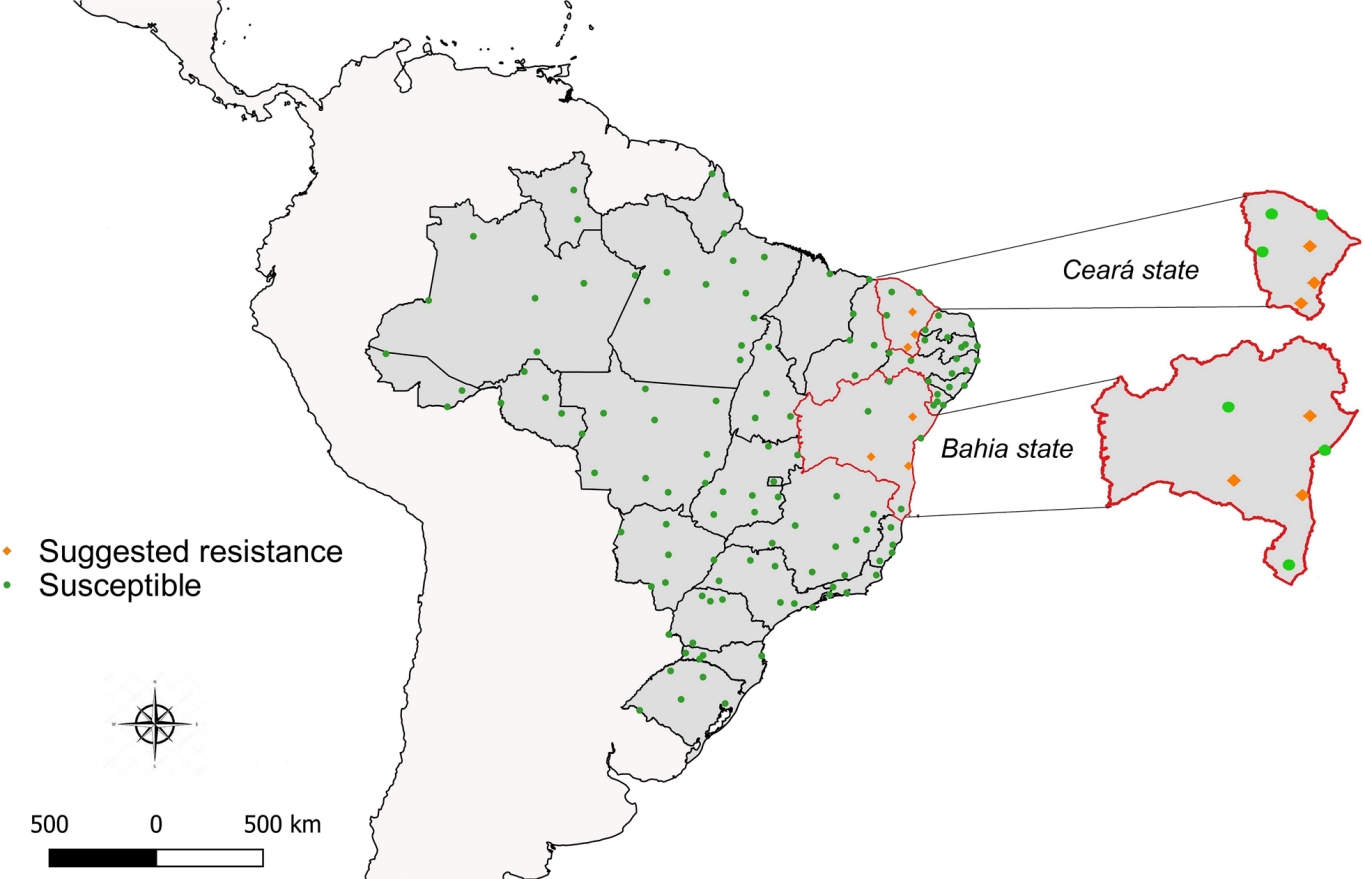
Map of Brazil displaying the results of the IGR pyriproxyfen resistance evaluation for *Aedes aegypti* populations, 2017–2018. Green circles or orange diamonds represent localities where populations were susceptible or suggested resistance (IE < 98%) was noted, respectively. The states of Bahia (BA) and Ceará (CE) are highlighted and the municipalities presenting suggested resistance populations are indicated

The dose-diagnostic (DD) obtained for pyriproxyfen was of 0.015 µg/l (Table 3). Among the 132 evaluated populations, six (4.5%) from the Brazilian northeastern cities of Itabuna, Brumado and Serrinha (Bahia), Quixadá, Icó, and Juazeiro do Norte (Ceará), presented EI < 98%, thus being subjected to DR tests to assess resistance levels (Table 2, Fig. 2). Resistance ratios (RR_50_ and RR_95_) were low in these populations, ranging between 1.07–1.97 (RR_50_) or 1.51–3.58 (RR_95_) (Table 4), indicating low resistance. Approximately 137,280 larvae were tested to perform all dose-diagnostic larval assays for the 132 populations, followed by DR assays in six populations that did not exhibit pyriproxyfen susceptibility.

**Table 3:** Dose-response bioassay to determine the pyriproxyfen diagnostic dose for *Aedes aegypti*, Rockefeller strain

EI_50_ (µg/l)^a^	CI_50_ (µg/l)^b^	EI_99_ (µg/l)^a^	CI_99_ (µg/l)^b^	Slope
0.06205	0.06012–0.06394	0.15589	0.14655–0.16733	5.8164

^a^EI_50_ and EI_99_: pyriproxyfen concentrations needed to inhibition of 50% and 99% adults emergence, respectively

^b^CI: confidence intervals

**Table 4:** Dose–response bioassays on *Aedes aegypti* populations resistant to pyriproxyfen in Brazil, 2017–2018

Region	State	Population/City	EI_50_ (µg/l)^a^ (CI)	EI_95_ (µg/l)^a^ (CI)	RR_50_^b^	RR_95_^b^	Slope	Resistance level^c^
		Rockefeller	0.0621 (0.0620–0.0639)	0.1190 (0.1137–0.1253)	1.00	1.00	5.81	–
Northeast	Bahia	Serrinha	0.1207 (0.0312–0.4665)	0.4257 (0.1711–1.0595)	1.95	3.58	3,00	Low
		Itabuna	0.1223 (0.0942–0.1588)	0.4056 (0.2776–0.5927)	1.97	3.41	3.16	Low
		Brumado	0.0666 (0.0510–0.0871)	0.3160 (0.2699–0.3699)	1.07	2.66	2.43	Low
	Ceará	Juazeiro do Norte	0.0835 (0.0498–0.1399)	0.2495 (0.1884–0.3304)	1.35	2.10	3.46	Low
		Quixadá	0.0900 (0.0800–0.0900)	0.2200 (0.2000–0.2400)	1.45	1.85	4.31	Low
		Icó	0.0700 (0.0600–0.0800)	0.1800 (0.1500–0.2200)	1.13	1.51	4.25	Low

^a^EI_50_ and EI_95_: inhibition of 50% and 95% adult emergence pyriproxyfen concentrations, respectively (CI: confidence intervals)

^b^RR_50_ and RR_95_: resistance ratios

^c^Resitance level: RR_95_ < 5.0: low; RR_95_ 5.0–10.0: moderate; RR_95_ > 10.0: high Mazzarri & Georghiou [26]

**Fig. 3:**
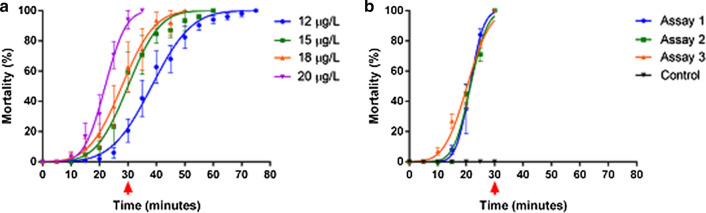
Determination of the malathion diagnostic-dose (DD) in *Aedes aegypti*, Rockefeller strain. **a** Mortality throughout the exposure period to bottles coated inside with different doses. **b** Three additional independent trials with DD set at 20 µg/ml, resulting in 100% mortality in 30 min. The red arrow highlights the 30 min mark

**Fig. 4:**
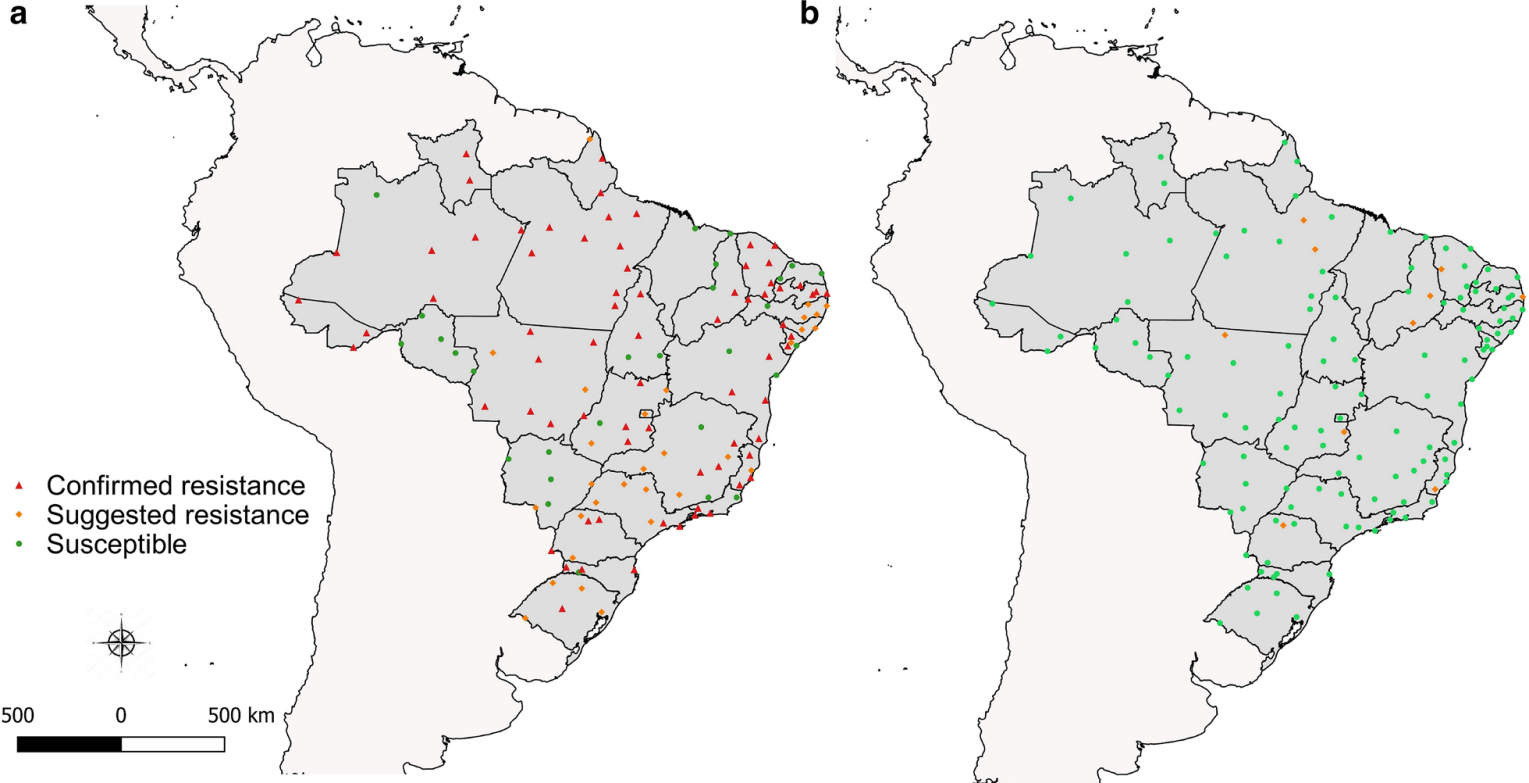
Map of Brazil displaying the results of the organophosphate malathion resistance evaluation for *Aedes aegypti* populations, 2017–2018. Diagnostic-dose tests employed a 20 µg/bottle (**a**) or 50 µg/bottle dose (**b**). Green circles, orange diamonds or red triangles represent localities where populations were considered susceptible, with suggested resistance or with confirmed resistance, respectively

The DD obtained for malathion under our laboratory conditions was of 20 μg/bottle (Fig. 3), 2.5-fold lower than the established WHO value (50 µg/bottle). In the 20 µg/bottle DD tests (Fig. 4a), 28 populations (21.4%) presented mortality above 98% (susceptible), 30 (22.9%) exhibited mortality between 90 and 98% (suggested resistance) and 73 populations (55.7%) displayed mortality below 90% (confirmed resistance). On the other hand, when exposed to 50 µg/bottle (Fig. 4b), most of the populations (121, 92.4%) were considered susceptible, and the remaining (10, 7.6%), as presenting “suggested resistance”, with mortality rates ranging from 90 and 98%. Approximately 131,000 *Ae. aegypti* female adults from 131 field populations were required for the malathion susceptibility testing. As noted in the map displayed in Fig. 4a, although localities with populations where resistance to 20 µg/bottle malathion was suggested are spread out throughout the country, the north region concentrates the highest percentage of resistant populations (71.9%).

## Discussion

The present study evidenced the feasibility of conducting an insecticide resistance monitoring action in a standardized and strongly coordinated manner, applying a model that may be of assistance in implementing national monitoring plans in other countries. A systematic literature review covering insecticide resistance data in *Ae. aegypti* field populations from Latin America and the Caribbean indicates that less than half of the countries in this region have published bioassay data between 2008 and 2018 [7]. In addition, the number of populations representing each national surveillance was generally rather low [7]. Susceptibility monitoring to temephos and deltamethrin carried out between 1999 and 2011 by the previous “National Network for Monitoring the Resistance of *Ae. aegypti* to Insecticides” generally evaluated between 25 and 74 populations every two years [17].

Out of all *Ae. aegypti* populations evaluated herein, 99.3% were classified as susceptible to the IGR pyriproxyfen. The six resistant populations were from the same geographical region (Northeast), in the states of Bahia (Itabuna, Brumado and Serrinha) and Ceará (Quixadá, Icó and Juazeiro do Norte), suggesting the emergence of localized pyriproxyfen resistance. Interestingly, some of these populations exhibited discrepant RR_50_ and RR_95_ values, suggesting a heterogeneous response within the population, as represented by low slope values (Table 4). These populations are likely experiencing an initial selection process, where only some individuals exhibit resistance so far. We hypothesized that this regionalization is related to differences in operational applications and the amount of applied insecticides, as well as due to population genetic background peculiarities, although no evidence to support this so far is available. It is noteworthy that *Ae. aegypti* populations from the Northeast presented the highest levels of temephos resistance in Brazil [9], as well lower residual effects in field assays, noted in populations from localities where high temephos RRs were previously described [27]. These data were collected before the introduction of pyriproxyfen use, suggesting cross-resistance. In the case of Itabuna, in the state of Bahia, simulated field trials carried out in 2015 demonstrated 100% pyriproxyfen efficacy within 30 days after application, albeit with a significant drop in the EI after 45 days [28]. Further investigations are required in order to better understand the mechanisms related to this trend.

We evidenced that the lowest malathion concentration able to kill 100% of Rockefeller females in 30 min was 20 µg/bottle, a 2.5-fold lower dose than that recommended by WHO in bottle assays (50 µg) [24]. No malathion-resistant populations (mortalities of less than 90%) were observed when the WHO DD 50 µg/bottle was employed, while 73 populations (55.8% of the total evaluated) were classified as resistant in the 20 µg/bottle exposure assays. The WHO-suggested DD is based on tests performed in reference laboratories and estimated from a variety of susceptible strains for resistance detection, seeking easy testing and reliability. This DD should be considered as a guide that may be refined for local situations whenever possible [29]. The local DD was more sensitive in the early discrimination of resistant individuals. This results in an interesting approach in identifying decreased susceptibility before reaching levels that may incur in loss of insecticide effectiveness in the field. The resistance monitoring programme in Brazil seeks to detect early susceptibility changes so that the applied product may be changed in a timely manner. Early detection would also permit management approaches enabling to more rapidly revert to the susceptible status of a population in cases where resistance is not so high.

The meaning of laboratory-observed resistance associated to product effectiveness under field conditions should be studied. Assessments conducted two decades ago had already reported *Ae. aegypti* resistance to malathion in northeastern Brazilian populations, when OPs were used to control both the larval (temephos) and adult (malathion) phases [17]. Insecticide selection against *Ae. aegypti* in Brazil followed the WHO criteria, also indicating that a product should be replaced in areas with a high RR (> 10.0) and with confirmed lack of efficacy in simulated field tests [11]. However, insecticide substitution takes an average of two years [2], since it depends on series of bureaucratic processes. Therefore, the time spent between the first detection of resistance in a laboratory bioassay and the effective change of the compound in the field has not been effective in precluding the spread of insecticide resistance. In order to avoid decreased insecticide effectiveness in the field, a more sensitive replacement criterion has been adopted since 2006. In this regard, changing the active ingredient of the insecticide is recommended in localities where mosquito populations present mortality rates below 70% in DD assays or with RR_95_ > 3.0, which occurs before the previous applied management criteria, of mortality rates below 80% in DD assays and RR_95_ > 10.0 [11]. Results for the state of São Paulo were the basis for this arrangement, where simulated field trials with temephos demonstrated failures in the control of *Ae. aegypti* in populations exhibiting RR_95_ > 3.0. PYs were ineffective in simulated field trials against populations with mortality rates below 70% in the DD in laboratory bioassays [30]. This was a very severe criterion, aiming to preserve resistance evolution or reverse it. Since no RR values > 5 for pyriproxyfen are observed in the country, IGR use may be continued, although the best scenario would be to apply another insecticide class in locations presenting suggested resistance.

Concerning adulticides, the situation is alarming, since there is only one available alternative to PY and to the OP malathion, i.e. the association of prallethrin with imidacloprid [31]. In the most recent national evaluation concerning PYs (2011 and 2012) high RRs for deltamethrin were observed throughout the country [8]. In addition, localities with higher numbers of dengue incidence in São Paulo were also those exhibiting higher levels of PY resistance, although these compounds were no longer being applied by governmental campaigns against *Ae. aegypti*. This is associated to the excessive use of insecticides in households, especially during arbovirus epidemic seasons, and PYs application against other urban vectors, as observed in an area where an intense campaign against *Leishmania* vectors was implemented [32]. The present study demonstrated resistance to malathion in most of the evaluated mosquito populations with the 20 µg/bottle DD. Therefore, chemical control against *Ae. aegypti* is crucially threatened in most Brazil territory, as long as no other alternative compound is available.

Emerging resistance to all the main classes of neurotoxic insecticide (CA, OC, OP and PY) has been detected in *Ae. aegypti* from the Americas, Africa and Asia [33]. The occurrence of susceptibility alterations concerning IGR, the most recently adopted class of insecticides, reinforces the importance of using integrated tools that can contribute to reduce the need for chemical vector control, modifying arbovirus transmission determinants, such as sustainable environmental management and education actions [34]. Lesser use of chemical insecticides reduces the risk of associated factors, such as ecological imbalances, secondary pest outbreaks and harmful effects to human health and to other non-target animals [35].

An alert is required concerning the high frequency of populations also comprising *Ae. albopictus* (59.8%). Our sampling was performed on the grounds of houses in urban territories, evidencing the significant expansion of this species in the country since its first record in 1986, in rural areas [36]. Further studies are recommended to better understand the role of *Ae. albopictus* in arbovirus transmissions in Brazil. In parallel, the monitoring of insecticide *Ae. aegypti* resistance should also consider *Ae. albopictus* populations.

Finally, the evaluation of all 146 planned populations was not possible, since some samplings were not carried out due to operational difficulties, while the laboratory maintenance of some populations was prevented by insufficient or inadequate egg preservation, hindering hatching. This limitation was minimized by providing the necessary material to all participants and preparing a video in order to standardize sampling and laboratory transport procedures.

## Conclusions

The challenge posed by vector resistance to different active ingredients available for their chemical control reinforces the importance of implementing Integrated Management Strategies, which prioritize mechanical control and educational actions, with the aim of decreasing the number of breeding sites [1, 2]. A well-structured mosquito insecticide resistance monitoring system is essential for a sustainable, integrated and effective plan based on chemical vector control strategies. We described the sampling and standardization activities of insecticide resistance monitoring tests for *Ae. aegypti* from 132 Brazilian localities between 2017 and 2018, discussing their results in the light of knowledge acquired since the first monitoring round carried out in 1999. We currently recommend the substitution of pyriproxyfen for an alternative larvicide class in areas where susceptibility changes were detected, in order to preserve the efficacy of this IGR. Regarding adulticides, resistance to malathion was as widespread in all Brazilian regions through laboratory-based DD assessments. Therefore, an alternative class of insecticide should be used to control adult mosquitos, also considering the previously noted history of pyrethroid resistance in Brazil. Resistance monitoring and the evaluation of new products must be performed continuously in locations that represent Brazil’s geographical, climatic and urban diversity.

## Data Availability

Data supporting the conclusions of this article are included within the article. The datasets required to reproduce the analyses and results presented herein are available from the corresponding author upon reasonable request.

## Abbreviations

BPU: Benzo-phenyl urea
B*ti*: *Bacillus thurigiensis*
CA: Carbamate
CDC: Centers for Disease Control and Prevention
DD: Diagnostic dose
DR: Dose-response
EI: Adult emergence inhibition
F1: First generation
F2: Second generation
FIOCRUZ: Oswaldo Cruz Foundation
IGR: Insect growth regulator
IOC: Oswaldo Cruz Institute
IR: Insecticide resistance
LAFICAVE: Laboratory of Physiology and Arthropod Control Vectors
LD: Lethal dose
LEnA: Laboratory of Applied Entomology
MoH: Ministry of Health
MoReNAa: National Network for Monitoring the Resistance of *Aedes aegypti* to Insecticides
PNCD: National Dengue Control Program
OP: Organophosphate
PY: Pyrethroid
RIDL: Release of insects with dominant lethality
RR: Resistance ratio
SIT: Sterile insect technique
SUCEN: Endemic Control Superintendence
WHO: World Health Organization
WP: Wettable powder
